# Short-Term Malaria Reduction by Single-Dose Azithromycin during Mass Drug Administration for Trachoma, Tanzania

**DOI:** 10.3201/eid2006.131302

**Published:** 2014-06

**Authors:** Stephen E. Schachterle, George Mtove, Joshua P. Levens, Emily Clemens, Lirong Shi, Amrita Raj, J. Stephen Dumler, Beatriz Munoz, Shelia West, David J. Sullivan

**Affiliations:** Johns Hopkins Bloomberg School of Public Health, Baltimore, Maryland, USA (S.E. Schachterle, L. Shi, A. Raj, D.J. Sullivan);; National Institute for Medical Research, Ubwari, Tanzania (G. Mtove);; Johns Hopkins University School of Medicine, Baltimore (J.P. Levens, E. Clemens, J.S. Dumler, B. Munoz, S. West)

**Keywords:** malaria, parasites, azithromycin, mass drug administration, single dose, prospective cohort study, trachoma, Tanzania

## Abstract

Single-dose mass drug administration of azithromycin (AZT) is underway to eliminate trachoma worldwide. Studies in Ethiopia showed a reduction in all-cause childhood deaths after administration. To examine the effect of single-dose AZ MDA on prevalent malaria infections in a large prospective cohort of children and parents in Dodoma Province, Tanzania, we quantified the temporal prevalence of malaria parasitemia by real-time PCR for 6 months after single-dose AZT. In the first month after treatment but not in subsequent months, *Plasmodium falciparum* infections were reduced by 73% (95% CI 43%–89%) in treatment versus control villages and differences remained significant (p = 0.00497) in multivariate models with village-level random effects. Genetic sequencing of *P. falciparum* ribosomal L4 protein showed no mutations associated with AZT resistance. AZT mass drug administration caused a transient, 1-month antimalarial effect without selecting for *P. falciparum* ribosomal L4 resistance mutations in a region with a 10-year history of treating trachoma with this drug.

## Abstract 2

This drug might be beneficial in areas to which malaria and trachoma are endemic.

Malaria can be treated or prevented with the broad-spectrum antimicrobial drugs tetracycline or azithromycin (AZT) (*1*). In vitro, AZT interferes with malarial parasite replication by targeting the unique apicoplast organelle of the parasite (*1*). AZT inhibits malarial parasite growth 10–fold every 48 hours, and the pharmacokinetics of AZT predict that it remains at concentrations high enough to limit parasite growth for >1 week (*2*). AZT might interfere with transmission by exoerythrocytic inhibition of parasite liver stages in humans and mice (*3*) and by interference with ookinete and sporozoite production in mosquitoes (*4*). Monotherapy with AZT is not typically used to treat malaria. However AZT is highly effective against *Chlamydia trachomatis*, the causative agent of trachoma, which causes blindness (*5*,*6*). Persons with malaria who live in trachoma-endemic regions may undergo repeated AZT therapy as part of the World Health Organization–sponsored global trachoma eradication program (*7*,*8*).

Data regarding the effects of AZ mass drug administration (MDA) on malaria are limited. In a cluster randomized trial in The Gambia, AZ MDA given in 3 doses (20 mg/kg) 7 days apart reduced malaria rates by half when measured at 1 time point in children 5–14 years of age (*9*). More recently, a 49% reduction in the odds of death (95% CI 29%–90%) was reported for children 1–9 years of age in an AZ MDA treatment group compared with controls in Ethiopia (*10*,*11*). Porco et al. suggested that reductions in malaria prevalence associated with AZT MDA might have contributed to observed decreases in overall deaths. (*10*).

For malaria treatment, a randomized clinical trial that compared AZT/artesunate with artemether/lumefantrine in Muheza, Tanzania, reported that the odds of treatment failure were 5 times greater (95% CI 3.3–11.4) in the group that received AZT/artesunate (*12*). The authors postulated that the AZT/artesunate showed treatment failure because MDA for trachoma in Tanzania could have led to localized *Plasmodium* spp. resistance to AZT (*12*). *Plasmodium* spp. drug resistance to AZT has not been documented in the field. However, in vitro selection for AZT resistance identified a G76V mutation among conserved active site amino acids at position 71–79 in *P. falciparum* apicoplast-encoded ribosomal protein L4 (*Pf*RpL4) (PFC10_API0043) (*1*.) A Cochrane meta-analysis report stated that “azithromycin’s future for the treatment of malaria does not look promising” (*13*) and cited studies in which AZT, although well tolerated, was inferior to tetracycline for malaria prophylaxis (*14*–*17*).

In the current study, we evaluated malaria prevalence in a cohort of 2,053 children and adults in central Tanzania to examine the effect of single dose AZ MDA on prevalent malaria infections. We also searched for *Pf*RpL4 mutations that might confer *P. falciparum* AZT resistance.

## Methods

### Study Site and Sampling

Study participants were from 8 rural agricultural villages in Dodoma Province, central Tanzania (*18*–*20*). The study period was January 12, 2009–July 21, 2009 and was coincidental with a period in which rainfall was <60% of the average amount (*21*,*22*). AZT was offered to all the residents of the treatment villages, including residents not part of the follow-up investigation. Four treatment villages were selected on the basis of trachoma prevalence >10% in children 1–9 years of age (trachoma prevalence 12%, 18%, 18%, and 14%), rather than by randomization (*18*,*19*), which was consistent with World Health Organization AZT MDA guidelines for trachoma control (*4*). Four control villages with lower trachoma rates (8%–10%) were chosen on the basis of geographic proximity.

A complete census was conducted in the villages> In each village, 130 families with children <5 years of age were randomly selected for follow-up prevalence blood sampling; 1 child and 1 adult were randomly selected from each family. Sample size estimations were based on prestudy estimates of a malaria prevalence of 10% in children <5 years of age in the Dodoma region (*23*). Follow-up fingerprick blood sampling was performed at baseline and at 1, 3, 4, and 6 months later. Children were divided into 3 subgroups that were sampled once at weeks 2 (group 1), 6 (group 2), and 8 (group 3). Contemporaneous to the monthly blood sampling, staff members from villages conducted an active surveillance program in which children were visited weekly and screened for axillary temperature and questionnaires were answered regarding fever, diarrhea, and respiratory disease (*18*,*19*).

Blood samples were collected on ProteinSaver903 (GE Healthcare, Pittsburgh, PA, USA) for analysis by real-time PCR for all participants at each scheduled monthly visit (*20*). When a fever (temperature ≥37.5°C) in adults and children was observed or when a caregiver reported a history of fevers in children during the weekly surveillance, a rapid diagnostic test (RDT) (Paracheck-Pf; Orchid Biomedical Systems, Goa, India) and thick and thin blood films were prepared. RDTs were performed by trained study staff and interpreted in the field at the time of sampling. Two experienced microscopists at the Amani Laboratory in Muheza, Tanzania, read the slides blinded to the PCR or RDT results. Discordant results were read by a third microscopist. Persons with positive results by RDT and all febrile children <5 years of age were treated with artemether/lumefantrine according to national guidelines and removed from later analysis.

Information on bednet ownership was obtained through standardized participant interviews. Latitude, longitude, and altitude of home locations were measured by using the GPSMAP 76 unit (Garmin, Olathe, KS, USA).

### Quantitative PCR

DNA extraction and real-time PCR are described elsewhere (*20*). Five 3-mm diameter punches, equivalent to 25 μL of whole blood, were removed from the filter papers. DNA was extracted by using a commercial 96-well kit (Promega, Fitchburg, WI, USA). The DNA was concentrated by glycogen acetate and acetate/ethanol precipitation and low-speed (3,000 × *g*) centrifugation for 30 min.

Multiplex real-time PCR was used to amplify the 18S *P. falciparum* ribosomal gene with a Cy5-labeled probe (*20*). Samples were processed in duplicate on manually loaded 384-well plates. A 40-cycle standard PCR protocol was used in a CFX 384 real-time PCR Detection System Thermocycler (BioRad, Hercules, CA, USA). Baseline relative fluorescence units (RFUs) were readjusted by using Bio-Rad CFX manager software. The real-time PCR system also detected *Borrelia* spp. reported by Reller et al*.* (*24*). The sensitivity and specificity of the real-time PCR versus that of RDT or microscopy were reported by Schachterele et al. (*20*). The real-time PCR detected 1–100 parasites/μL and showed the highest sensitivity in latent class analysis on febrile study patients. For the monthly surveys, microscopic analysis was not performed for the 8,711 samples for which real-time PCR was conducted. The RFU cutoff used was greater than that for >50 negative blood samples from control patients at Johns Hopkins University. The cutoff of 650 RFUs for the last cycle of the highest real-time PCR replicate had a specificity of 100% for samples from the control group at Johns Hopkins University and a specificity of 94% for samples from febrile patients in Tanzania; microscopy was used as a reference method (*20*).

### Single-Nucleotide Polymorphism Analysis of *Pf*RpL4 Gene for AZT Resistance

Blood samples from treatment and control villages that had higher parasite densities by real-time PCR and microscopy were subjected to PCR amplification and whole gene fragment cloning into the pCR2.1 plasmid. Amplification and cloning were followed by full *Pf*RpL4 gene DNA sequencing of multiple bacterial clones for each patient and examination of mutations anywhere in this gene (*1*).

### Statistical Analysis

Data were analyzed from the perspective of an intention to treat in which participants from AZ MDA–treated villages who refused treatment were classified with those who accepted AZT as members of treatment villages. Univariate ratios were used to compare prevalence proportions in treated versus untreated villages. CIs for ratios that compared proportions of prevalent infection in treated versus untreated villages were estimated by using 2-sided exact binomial tests.

Maps were used to display geospatial patterns in infection prevalence and malaria clustering over time (Google Maps, Mountain View, CA, USA, and Quantum Geographic Information System Open Source Geospatial Foundation Project (www.qgis.org/en/site/). Geographic coordinates of *P. falciparum*–positive and –negative blood samples were projected into kilometers, and locations were smoothed by using quadratic kernel intensity estimation. Kernel intensity estimators predict spatial intensity at unsampled points from nearby sampled points by using a quadratic function to heavily weight the nearest measured points and de-emphasize points sampled at greater distances. Weighted integrals were used to estimate intensity smoothing parameters for quadratic functions, assuming a 2-dimensional (i.e., spatial), stationary, isotropic point pattern process. Spatial odds ratios (ratio of kernel intensities from positive blood samples to kernel intensities from negative blood samples) (*25*), were determined for *P. falciparum*.

To examine the effect of AZT MDA while adjusting for malaria clusters observed on maps, we used multivariate logistic models with random effects to compare odds of prevalent malaria infection for AZT MDA treatment villages with odds for control villages. Random intercepts were fit to account for the multilevel or hierarchical design that nested persons within villages. This model enabled accurate statistical inference by adjusting CIs and p values for *P. falciparum* clusters in villages, and this inference was apparent in prevalence maps (*25*). Residual spatial autocorrelation was assessed by using variograms, and residual temporal autocorrelation was eliminated by restricting models to 1 sampling interval (*26*). Multiple models that examined the effect of AZT MDA on prevalent malaria were fit to control for confounding by other drivers of malaria prevalence and to address the robustness of standard errors to potential spatial residual autocorrelation. Altitude and self-reported bednet ownership were held constant to control for confounding in the multivariate model. A priori, we believed any AZT MDA effect would be most perceptible between month 1 and baseline.

To distinguish between the effect of AZT on prevalence and incident infections, we performed a sensitivity analysis for only *P. falciparum* infections that had been preceded by a negative *P. falciparum* test result in the previous sampling interval. These data also underwent a second sensitivity analysis that included participants treated with artemether/lumefantrine who had been removed from the previous analyses.

Data management and analysis were conducted by using R statistical software (*27*). R with the SPLANCS package was used for kernel intensity estimation (*28*), and the LME4 package was used for the random effects model (*29*). All R code is available upon request.

## Results

### Characteristics of Study Population

**Figure 1:**
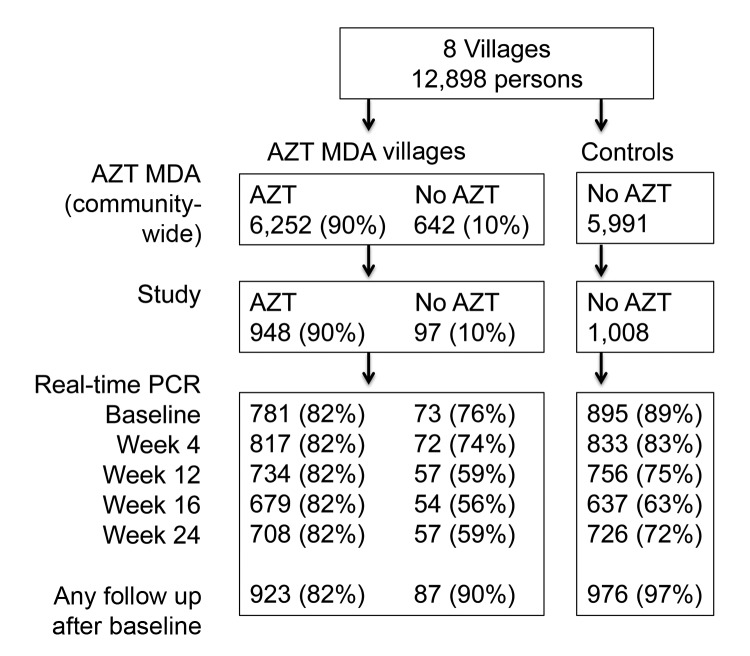
Flowchart of participants in study of short-term malaria reduction by single-dose azithromycin (AZT) during mass drug administration (MDA) for trachoma, Tanzania, January 12–July 21, 2009. AZT MDA (village-wide) and study panels show that 90% of persons who were intended to receive AZT received this drug. Total study participants with ≈1,000 in each group, shown in the study panel, contributed samples that are shown in the real-time PCR panel at each sampling time. Percentages in the real-time PCR panel used values from the study panel as the denominator.

The study site in the Kongwa District of Tanzania included 8 villages and 12,898 persons. Four villages were chosen on the basis of increased prevalence of trachoma (>10%) in children 1–9 years of age. Excluded were 34 of 66 villages that had received AZT MDA in the previous year. In the 4 villages that received AZT MDA, 6,252 persons received AZT and 642 did not receive AZT. A census in untreated control villages identified 5,991 persons. These villages were chosen because of geographic proximity to treatment villages. In each village, pairs of a parent and a child <5 years of age were randomly chosen for inclusion (1,045 persons in the AZT MDA treatment group and 1,008 persons in the control group for follow-up) (Figure 1). Blood samples for real-time PCR testing were collected from participants at baseline and at months 1, 3, 4, and 6 (4,437 patient-time samples from AZT MDA treatment villages and 4,274 patient-time samples from control villages). At any follow-up period after baseline, 1,010 (97%) study participants in AZT MDA villages who received AZT and 976 (97%) study controls were sampled for real-time PCR at least once. At baseline, treatment and control villages reported antimalarial drug use, latrine access, education (based on the highest level of education attained by the father), and home elevations. However, self-reported bednet ownership and fever histories were higher in treatment villages than in control villages (p<0.05) (Table 1).

**Table 1:** Comparison between azithromycin MDA treatment and control participants at baseline, Tanzania, January 12–July 21, 2009*

Variable	MDA treatment group	MDA control group
Bednet ownership, yes/no†	457/725 (63)	328/789 (42)
History of fever, yes/no†	26/813 (3)	10/869 (1)
Malaria medication, yes/no	157/814 (19)	161/869 (19)
Latrine access, yes/no	297/981 (30)	276/971 (28)
Education of head of household, yes/no	4.2 (3.41)	3.2 (3.46)
Altitude, m	1,206 (32.89)	1,200 (45.99)

*Values are no. positive/no. tested (%) for categorical measures or mean (SD) for continuous measures. MDA, mass drug administration. †p<0.05 by χ^2^ test.

### Univariate Analysis by Time

**Figure 2:**
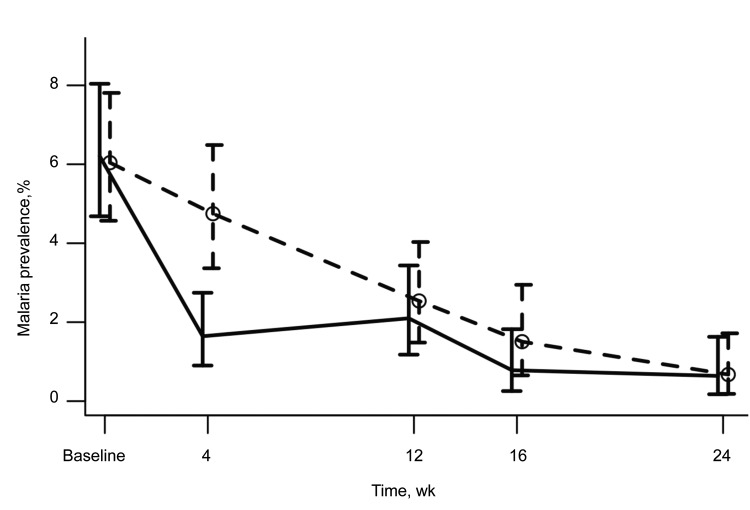
Effect of azithromycin (AZT) mass drug administration (MDA) in treatment and control villages by time in study of short-term malaria reduction by single-dose AZT during MDA for trachoma, Tanzania. January 12–July 21, 2009. Proportions of real-time PCR prevalent *Plasmodium falciparum* infections are shown in participants from treatment villages (solid line) and control villages (dashed line and circles). Error bars indicate 95% CIs from exact binomial tests.

**Figure 3:**
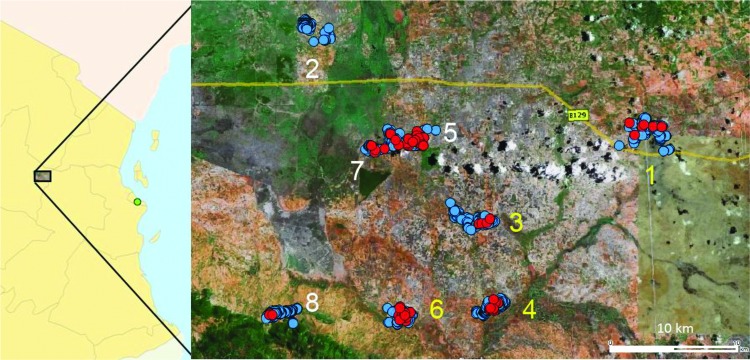
Study villages and location of malaria cases at baseline, Tanzania, January 12–July 21, 2009. Study site included 8 rural villages in central Tanzania, Dodoma Province. Four azithromycin (AZ) treatment villages in southeastern Tanzania are indicated by yellow numbers 1, 3, 4, and 6. Four nearby control villages in northwestern Tanzania are indicated by white numbers 2, 5, 7, and 8. The background map was extracted from Google Earth (Google, Mountain View, CA, USA). Households are indicated by blue dots, malaria cases at baseline are indicated by red dots, and Dar es Salaam (capital) is indicated by the green dot.

Overall, the proportion of *P. falciparum*–infected participants was highest (6%) at baseline. Infections decreased sharply in treated villages and gradually in control villages throughout follow-up period (Figure 2). At the baseline evaluation, 6% (53/854) of participants from AZT MDA treatment villages were positive for *P. falciparum* compared with 6% (54/894) of participants from AZT MDA control villages. No differences in odds of infection were observed between control and treatment villages (odds ratio 1.03, 95% CI 0.68–1.55) (Figure 3, Table 2).

**Table 2:** Univariate association between AZT MDA and *Plasmodium falciparum* malaria prevalence, Tanzania, January 12–July 21, 2009*

Time	No. negative	No. positive	OR (95% CI)
Baseline			
AZT–	840	54	1.03 (0.68–1.55)
AZT+	801	53	
Month 1			
AZT–	742	37	0.34 (0.17–0.64)†
AZT+	837	14	
Month 3			
AZT–	653	17	0.83 (0.38–1.77)
AZT+	700	15	
Month 4			
AZT–	523	8	0.52 (0.13–1.81)
AZT+	632	5	
Month 6			
AZT–	589	4	0.95 (0.18–5.12)
AZT+	621	4	

*AZT, azithromycin; MDA, mass drug administration; OR, odds ratio. †p<0.001.

By month 1, *P. falciparum* prevalence in AZT MDA treatment villages decreased to 2% (14/851) and *P. falciparum* prevalence in AZT MDA control villages decreased to 5% (37/779). The odds ratio for AZ MDA treated villages compared with control villages was 0.34 (95% CI 0.17–0.64), which is consistent with a 66% reduction in the odds of *P. falciparum* infection in AZT MDA treatment participants compared with AZT MDA control participants. Beyond month 1, the association between of AZT MDA and reduced prevalence of malaria infection decreased. However, overall rates of *P. falciparum* infection decreased below levels for which reliable inferences could be made.

**Figure 4:**
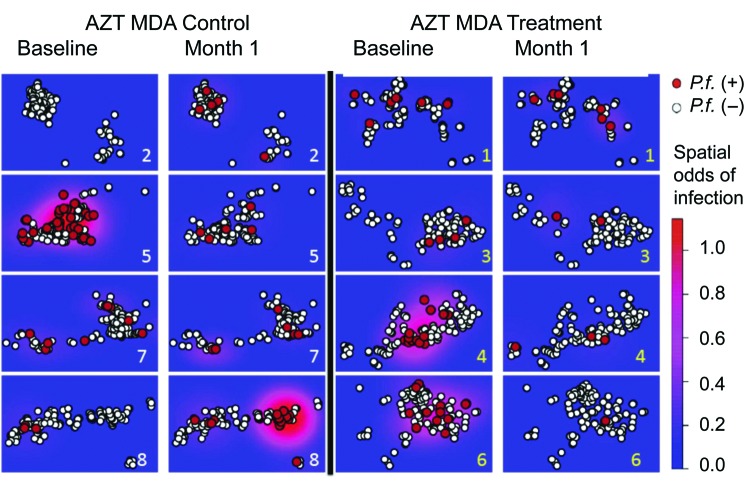
Effect of azithromycin (AZT) mass drug administration (MDA) in treatment and control villages over space and time, Tanzania, January 12–July 21, 2009. AZT MDA control villages (2, 5, 7, and 8) are shown on the left, and AZT MDA treatment villages (1, 3, 4, and 6) are shown on the right. Survey periods at baseline and month 1 are shown within treatment and control groups. Baseline infection and prevalent infections at month 1 (red circles) and negative tests (white circles) are shown. Spatial odds of *Plasmodium falciparum* (*P.f*.) infection are shown in areas of high odds of infection (red) and areas of low odds of infection (blue). Ln kernel smoothers were used to show spatial odds.

A village level comparison of changes in malaria prevalence for AZT MDA treatment villages versus prevalence in control villages indicated that 1 village in each group had no change in malaria prevalence, and 3 AZ MDA treatment villages had a significant (p<0.05, by Fisher exact test) decrease in malaria prevalence (p = 0.489, p = 0.0002, and p = 0.0005) and 2 in the control group had significant (p = 0.0008 and p = 0.001) increases. Village 5 in the control group had a significant (p = 0.0001) decrease (Figure 4; Technical Appendix Table 1).

In a subgroup of 200 children examined for malaria infection at weeks 2, 6, and 8, the malaria prevalence of 2%–5% was lower than the 10% expected (*23*) Thus, reliable prevalence comparisons were not relevant. During the study, febrile patients were tested by RDT and microscopy. During the critical period of 5–42 days after AZT MDA, 46 and 48 children in the treatment and control villages, respectively, were evaluated for fever. Among febrile children, only 5 children from treatment villages and 3 children from control villages were positive by real-time PCR.

### Multivariate Analysis

At month 1, reductions in *P. falciparum* malaria prevalence were observed by logistic regression models that controlled for differences in self-reported bednet ownership and home altitude (Table 3) and included village level random effects. The odds of *P. falciparum* infection were 63% (95% CI 28%–81%) but less in treated villages than in control villages after adjusting for bednet ownership and home altitude in a model with village-level random intercepts. Because self-reported history of fever, malaria medication, latrine access, and education of the head of the household were not associated with malaria prevalence and did not appreciably alter the interpretations of the multivariate models, they were not included in the reported estimates. As was evident in univariate analysis, no differences in malaria rates existed at baseline, and the association between AZT MDA and reduced malaria rates decreased after the first month of follow up.

**Table 3:** Multivariate association between AZ MDA and *Plasmodium falciparum* prevalence with village-level random effects, Tanzania, January 12–July 21, 2009*

Time	AZT MDA treatment, yes/no	Bednets, yes/no	Altitude, m
Baseline	1.16 (0.725–1.849)	0.75 (0.483–1.158)	0.99 (0.984–0.996)
Month 1	0.37 (0.188–0.743)†	0.78 (0.418–1.439)	1.01 (1.002–1.017)
Month 3	0.70 (0.922–1.64)	1.55 (0.662–3.65)	1.00 (0.992–1.012)
Month 4	0.62 (0.078–4.919)	0.40 (0.095–1.673)	1.02 (0.997–1.044)
Month 6	0.50 (0.074–3.347)	1.54 (0.235–10.094)	0.99 (0.966–1.016)

*Values are odds ratio (95% CI). AZT, azithromycin; MDA, mass drug administration. †p<0.005.

Spatial information on home locations enabled a thorough examination of residual spatial autocorrelation. A total of 51% (19/37) of *P. falciparum* infections at month 1 occurred in control village 8 (Figure 4), which raised concerns that residual spatial autocorrelation artificially decreased CIs from the reported models despite use of the random effects model. However, a variogram of standardized residuals from the model suggested that residual spatial autocorrelation was appropriately controlled by the village level random effects. The data were reanalyzed with incident infections that had been preceded by a negative real-time PCR result for *P. falciparum*; results did not change appreciably. We also observed an age-independent decrease in malaria prevalence at month 1 for persons 1–10 years of age (univariate analysis) and for persons >10 years of age (univariate analysis and multivariate analysis) (Technical Appendix Table 2). 

### Analysis of *Pf*RpL4 Mutations Associated with Azithromycin Resistance

Sequencing of full-length *P. falciparum* ribosomal L4 protein was performed for samples from 12 patients. We did not find evidence of single-nucleotide polymorphisms (SNPs) conferring AZT resistance in amino acid region 71–79 (*1*). A synonymous SNP at position K36 was found in all bacterial plasmid clones from samples of 1 patient and in a *P. falciparum*–positive control patient who was not from Tanzania. A single plasmid clone from a sample of participant from an AZT MDA control village contained a nonsynonymous SNP in the active site of the *Pf*RpL4 gene at A78S. However, 17 other plasmid clones from the same blood sample did not contain the mutation, which suggested the aberrant sequence resulted from a Taq polymerase error during plasmid cloning of the *Pf*RpL4 gene. Moreover, restriction enzyme *Hin*dIII, which was specific for the nonsynonymous SNP at A78S, did not digest multiple PCR amplicons from the original patient blood sample, which further suggested that the SNP on the *Pf*RpL4 gene was present at a low concentration or may have been an artifact of the Taq polymerase amplification. A78S has not been identified in other mutant bacteria associated with AZT resistance (*1*). Eighteen other nonsynonymous mutations were found in samples from participants from AZT MDA treatment villages, but these mutations were not in conserved amino acid regions for *Pf*RpL4 associated with AZT resistance in bacteria or the selected *P. falciparum* (except for an I39S were single occurrences among multiple clones from a single patient). Four synonymous SNPs were also detected (Table 4).

**Table 4:** Resistance markers for cloned sequences of *Plasmodium falciparum* ribosomal PfRp14 protein gene, Tanzania, January 12–July 21, 2009*

Patient ID	Time from azithromycin treatment, d	Parasites/μL by light microscopy	Parasites/μL by real-time PCR	RDT result	No. plasmid clones with WT sequence	Clones with synonymous SNP (no.)	Clones with nonsynonymous SNP (no.)	No. plasmids sequenced
1	30	31,200	5,378	+	7	L112L (1)	0	8
2	30	ND	3,177	ND	3	0	0	3
3	30	ND	224.9	ND	0	K36K (4)	0	4
4	67	151,000	126,506	+	2	0	0	2
5	69	74,400	55,850	–	1	S85S (1)	0	2
6	80	49,160	16,410	+	4	0	I38V(1), N109S (1), N166D (1)	7
7	82	1,840	679	+	1	0	F114L (1)	2
8	94	26,760	11,030	–	1	0	T56I (1)	2
9	127	0	51,075	+	3	0	0	3
10	132	ND	22,774	ND	0	0	I39S (1), S65N (1)	4
11	NT	0	69,718	+	13	0	A78S (1),† N134D (1), K100R and I126T (1), N166S (1), Y20H, K100%, and I139T (1)	18
12	NT	11,160	2,495	+	2	0	0	2
Control	NT			ND	2	0	0	2
Control	NT			ND	2	0	0	2

*ID, identification; RDT, rapid diagnostic test; WT, wild type; SNP, single-nucleotide polymorphism; +, positive; ND, not done; –, negative; NT, not treated.  †An A78S nonynonymous SNP in region associated with azithromycin resistance that was not present in other clones.

## Discussion

AZT MDA was associated with a minimum reduction of 66% in odds of *P. falciparum* infection compared with odds of reduction for controls in the first month after drug administration, as shown by univariate and multivariate models on an individual level. Beyond 1 month, prevalence in treatment and control groups decreased, and there was no difference in prevalence between treatment and control groups. In addition, no mutations were detected in samples from treated persons who had parasitemias after drug treatment in the only gene associated with in vitro selection of AZT resistance. However, these results show more modest effects than those of other studies of AZT MDA, which reported large reductions in malaria (*9*) and that AZT MDA protection against malaria contributed to broader decreases in illness and death (*10*).

Because our data were analyzed from the perspective of an intention to treat, participants who did not receive AZT but lived in villages that received AZT MDA were classified with those who received AZT. The intention-to-treat analysis could have biased reported results toward the null because persons in AZT MDA treatment villages who benefited from AZT might have been more likely to become infected (Technical Appendix Tables 3, 4). Because intention-to-treat analysis would probably bias results toward the null, we believe that it was a conservative analytic assumption.

An additional potential confounder is that all febrile children <5 years of age and adults who were positive for *P. falciparum* by RDT were treated according to national and Integrated Management of Childhood Illness guidelines with artemether/lumefantrine, which has a lingering 4-week prophylactic effect on parasitemia. However, inclusion of artemether/lumefantrine–treated participants did not affect the relevant proportions of malaria infection at month 1 (Technical Appendix Table 5).

AZT treatment occurred at the village level, which might have exacerbated similarities in malaria risk among participants in the same village and created village-level clustering (*25*). Clustering within villages can bias results if it is not addressed in the statistical models (*30*). Village-level random effects were used to adjust variance estimates for within-village clustering and provide valid multilevel statistical inferences, given the clustering inherent in the study design (*20*,*31*). We argue that a relevant comparison is between the proportion of AZT MDA–treated and control villages, rather than a grouping of 4 villages in each AZT MDA intervention and control villages. Grouping findings into only 8 units on a village level does not produce relevant results and was not the design of the study.

AZT MDA was conducted occurred according to the WHO SAFE (surgery, antibiotics, facial cleanliness, and environmental changes) trachoma control strategy, and assigning treatment to villages by randomization would have been unethical because of the established effectiveness of this strategy in the study region. AZT MDA was examined from an observational perspective with the longitudinal cohort. We sought to minimize lack of comparability by selecting villages from a similar geographic region and by controlling for bednet ownership and altitude in multivariate analysis. We believe that residual confounding and bias are minimal because similar *P. falciparum* infection prevalence rates were observed at baseline and at months 3–6 after the effect of the AZT MDA had decreased.

In Ethiopia, Porco et al*.* reported a 49% (95% CI 10%–71%) decrease in odds of death in a randomized trial of AZ MDA villages compared with control villages that received a single dose of AZ (20 mg/kg to ≤1 g) (*10*). AZT protection from *P. falciparum* infection might have contributed to the overall decrease in deaths. Although the effect of AZT MDA on prevalent infections was strong, most infections observed were asymptomatic. The study in Ethiopia also had 3 dosing strategies (annual, biannual, and quarterly), which resulted in two thirds of study participants receiving more annual doses than used in the study in Tanzania. Our data provide only weak evidence that a reduction in malaria infection contributed to the reduction in deaths reported in Ethiopia.

At the study site, we did not find evidence of *P. falciparum* AZT resistance markers on a gene previously implicated in AZT resistance in vitro. We also observed a short-lived but major reduction in malaria. AZT MDA does not produce *Plasmodium* spp. drug resistance probably because other drugs with different mechanisms of action are used to treat malaria. This study might provide an optimistic note for AZ MDA planners and those living in areas in developing nations to which malaria and trachoma are endemic.

Technical Appendix Alternative analysis of baseline and month 1 comparisons for short-term malaria reduction by single-dose azithromycin during mass drug administration for trachoma, Tanzania, January 12–July 21, 2009.
