# Supplementary material for: Short-Term Malaria Reduction by Single-Dose Azithromycin during Mass Drug Administration for Trachoma, Tanzania
Source: Emerg Infect Dis. 2014 Jun;20(6):941–9. doi: 10.3201/eid2006.131302 (PMC4036785; doi:10.3201/eid2006.131302)
Supplement: Technical Appendix — Alternative analysis of baseline and month 1 comparisons for short-term malaria reduction by single-dose azithromycin during mass drug administration for trachoma, Tanzania, January 12–July 21, 2009. [file 13-1302-Techapp-s1.pdf]

Article DOI: http://dx.doi.org/10.3201/eid2006.131302

Short-Term Malaria Reduction by Single-Dose Azithromycin during Mass Drug Administration for Trachoma, Tanzania

Technical Appendix

Alternative analysis of baseline and month 1 comparisons for short-term malaria reduction by single-dose azithromycin during mass drug administration for trachoma, Tanzania, January 12–July 21, 2009

Technical Appendix Table 1. Village-level comparisons of baseline with month 1 for AZT MDA, Tanzania, January 12–July 21, 2009*

		18S ribosomal gene of *Plasmodium falciparum*			
Village	AZT MDA	Time	No. patients negative	No. patients positive	p value
1	Yes	Baseline	225	7	
	Yes	Month 1	183	6	1
3	Yes	Baseline	230	5	
	Yes	Month 1	193	0	0.0489
4	Yes	Baseline	203	23	
	Yes	Month 1	162	2	0.0002
6	Yes	Baseline	143	18	
	Yes	Month 1	111	1	0.0005
2	No	Baseline	234	0	
	No	Month 1	186	6	0.008
5	No	Baseline	190	40	
	No	Month 1	155	4	0.0001
7	No	Baseline	199	11	
	No	Month 1	148	7	0.81
8	No	Baseline	217	3	
	No	Month 1	155	19	0.001

*AZT, azithromycin; MDA, mass drug administration.

Technical Appendix Table 2. Effect of AZT MDA by age, Tanzania, January 12–July 21, 2009*

		18S ribosomal gene of *Plasmodium falciparum*	
Time	Treatment	No. patients negative	No. patients positive
Prevalence in persons 1–10 y of age			
Baseline	No AZT	357	26
	AZT	346	22
Month 1	No AZT	319	14
	AZT	355	5†
Prevalence in persons >10 y of age			
Baseline	No AZT	403	23
	AZT	383	27
Month 1	No AZT	356	21
	AZT	415	9‡

*AZT, azithromycin; MDA, mass drug administration.
†Significantly different (p = 0.0338) from control in univariate model.
‡Significantly different (p = 0.0142) from control in univariate and multivariate models when controlling for altitude, rainfall, and village-level random effect.

Technical Appendix Table 3. Effect of AZT treatment on an individual level for untreated persons who lived in in a village that received AZT and who then moved to a village that did not receive AZT, Tanzania, January 12–July 21, 2009*

Time	Individual treatment	18S ribosomal gene of *Plasmodium falciparum*		No. patients given AL	OR (95% CI) for patients given AL	OR (95% CI) for patients not given AL
		No. patients negative	No. patients positive			
Baselne	No AZT	909	58	0	1.05 (0.69–1.58)	1.05 (0.69–1.58)
	AZT	732	49	0		
Month 1	No AZT	811	38	54	0.36 (0.18–0.67)†	0.36 (0.18–0.70)†
	AZT	768	13	36		
Month 3	No AZT	704	20	89	0.81 (0.41–1.61)	0.65 (0.29–1.41)
	AZT	649	12	73		
Month 4	No AZT	571	10	110	0.37 (0.09–1.25)	0.29 (0.05–1.15)
	AZT	584	3	92		
Month 6	No AZT	640	4	139	1.11 (0.25–4.83)	1.12 (0.21–6.06)
	AZT	570	4	134		

*AL, artemether/lumefantrine; OR, odds ratio; AZT, azithromycin.
†p = 0.0015.

Technical Appendix Table 4. Effect of AZT treatment for patients who did not receive AZT (herd effect), Tanzania, January 12–July 21, 2009*

Time	Village treatment	18S ribosomal gene of *Plasmodium falciparum*		No. patients given AL	OR (95% CI) for patients given AL	OR (95% CI) for patients not given AL
		No. patient negative	No. patients positive			
Baseline	No AZT	840	54	0	0.90 (0.23–2.556)	0.90 (0.23–2.56)
	AZT	69	4	0		
Month 1	No AZT	742	37	52	0.27 (0.01–1.653)	0.29 (0.01–1.78)
	AZT	69	1	2		
Month 3	No AZT	653	17	86	2.04 (0.38–7.21)	2.26 (0.41–8.18)
	AZT	51	3	3		
Month 4	No AZT	523	8	106	2.68 (0.28–13.42)	2.72 (0.27–14.15
	AZT	48	2	4		
Month 6	No AZT	589	4	133	0 (0–14.11)	0 (0–17.87)
	AZT	51	0	6		

*AL, artemether/lumefantrine; OR, odds ratio; AZT, azithromycin.

Technical Appendix Table 5. Comparison of persons given azithromycin with untreated persons, by village level, Tanzania, January 12–July 21, 2009*

Time	Village treatment	18S ribosomal gene of *Plasmodium falciparum*		No. patients given AL	OR (95% CI) for patients given AL	OR (95% CI) for patients not given AL
		No. patients negative	No. patients positive			
Baseline	No AZT	840	54	0	1.03 (0.68–1.55)	1.03 (0.68–1.55)
	AZT	801	53	0		
Month 1	No AZT	742	37	52	0.33 (0.17–0.62)†	0.34 (0.17–0.64)†
	AZT	837	14	38		
Month 3	No AZT	653	17	86	0.96 (0.48–1.89)	0.82 (0.38–1.77)
	AZT	700	15	76		
Month 4	No AZT	523	8	106	0.58 (0.17–1.82)	0.52 (0.13–1.81)
	AZT	632	5	96		
Month 6	No AZT	589	4	133	0.95 (0.22–4.14)	0.95 (0.18–5.12)
	AZT	621	4	140		

*AL, artemether/lumefantrine; OR, odds ratio; AZT, azithromycin.
†p = 0.0003.
